# Transesophageal Echocardiography‐Related Complications During Mitral Valve Repair in Dogs

**DOI:** 10.1111/jvim.70037

**Published:** 2025-03-17

**Authors:** Kentaro Kurogochi, Arane Takahashi, Yasuyuki Nii, Tomoya Suzuki, Masashi Mizuno, Masami Uechi

**Affiliations:** ^1^ JASMINE Veterinary Cardiovascular Medical Center Yokohama Kanagawa Japan; ^2^ Department of Clinical Sciences North Carolina State University, College of Veterinary Medicine Raleigh North Carolina USA

**Keywords:** dog, mitral valve plasty, mucosal lesion, myxomatous mitral valve disease, narrowband imaging

## Abstract

**Background:**

Transesophageal echocardiography (TEE) is an indispensable modality in cardiac surgery; however, the complications associated with its use in veterinary clinical settings remain unclear.

**Hypothesis/Objectives:**

To investigate the complications associated with TEE probe manipulation during mitral valve repair in dogs and identify the risk factors for new mucosal injuries.

**Animals:**

This prospective study evaluated 60 client‐owned dogs that underwent TEE to support mitral valve repair.

**Methods:**

Esophageal endoscopy was performed twice—once after anesthesia induction and once before extubation—to assess esophageal mucosal injuries during the procedure. The type of injury was classified as ‘complex’ (intramural hematoma and mucosal laceration), ‘minor’ (petechiae and ecchymosis), or ‘minute’ (visible only on narrowband imaging) lesions. During the surgery, TEE was performed three times. Hemodynamics were evaluated before the initial TEE insertion and immediately after it was removed.

**Results:**

Of the 60 dogs, newly detected mucosal lesions were observed in 20 dogs and classified as ‘minor’ in four and ‘minute’ in 16 dogs. These ‘minute’ lesions were not visible with conventional endoscopy, and no ‘complex’ lesions were identified. No significant factors were associated with the presence of mucosal lesions. After TEE insertion, systolic blood pressure decreased from 95 ± 13 mmHg to 92 ± 11 mmHg (*p* = 0.008), and heart rate decreased from 128 ± 25 bpm to 123 ± 24 bpm (*p* < 0.001).

**Conclusions and Clinical Importance:**

Intraoperative TEE results in a low incidence of esophageal mucosal injuries. Hemodynamic changes could occur during TEE manipulation, underscoring the need for close monitoring.

**Abbreviations**
IQR: interquartile range
MVR: mitral valve repair
NBI: narrowband imaging
SD: standard deviation
TEE: transesophageal echocardiography

## 1 Introduction

Transesophageal echocardiography (TEE) is a crucial modality in cardiac surgery in dogs. It is particularly useful for patent ductus arteriosus occlusion and balloon valvuloplasty for pulmonary valve stenosis and is also valuable for the diagnosis and detailed evaluation of less common and complex congenital heart diseases [1, 2, 3, 4]. In addition to catheter intervention, it is an important technique in open‐heart surgery. Perioperative TEE is a key diagnostic modality in human mitral valve surgery [5]. It offers comprehensive insights into valve anatomy, lesion severity, and ventricular performance, aiding surgeons in selecting the most appropriate surgical approach and assessing preoperative risks [6]. Additionally, TEE is used intraoperatively to monitor the progress of the surgery, allowing for adjustments to the initial surgical plan as needed. It is also invaluable for evaluating surgical outcomes and promptly diagnosing potential complications during the immediate postoperative period [7, 8]. Perioperative TEE influences surgical plans in 10%–25% of human mitral valve surgery [6]. Open‐heart surgery techniques in dogs, like those in humans, also require TEE for procedural planning and intraoperative support [9, 10, 11, 12, 13].

TEE is performed to support the surgery by confirming de‐airing as the patients come off cardiopulmonary bypass, providing immediate assessment of surgical results, and monitoring ventricular function and hemodynamic status intraoperatively [14]. Additionally, it offers high‐resolution images during mitral valve repair (MVR) in dogs [9, 15], which is a potential curative treatment option for dogs with advanced‐stage myxomatous mitral valve disease. In humans, although TEE is a relatively safe imaging modality with a low incidence of complications, the rates of adverse events are higher in pediatric patients than in adults owing to their smaller size [16]. To minimize the complications associated with TEE in pediatric patients, recommendations include selecting probes based on size and careful probe insertion and manipulation [17]. Dogs are similar to children in that they generally have smaller body sizes. A veterinary clinical study on TEE, focusing on the diagnosis and interventional procedures for congenital heart disease, reported only a few mild complications, even in small‐breed dogs (body weight < 4 kg) [18]. We assumed that small breed dogs with cardiac enlargement might compress the thoracic space and have a shorter distance from the heart to the vertebra [19], potentially increasing the risk of TEE‐related complications. However, the complications of TEE related to open‐heart surgery in dogs remain unclear. During the MVR procedure, heparinization is required to manage cardiopulmonary bypass [15], which carries a risk of bleeding. Dogs with MMVD often exhibit specific characteristics, such as advanced age, being small‐breed, and cardiac enlargement. Additionally, they could have reduced forward cardiac output due to severe mitral regurgitation. Therefore, evaluating potential adverse events in this dog population is particularly important.

Thus, this study aimed to evaluate the incidence and types of complications following TEE probe manipulation during MVR in dogs and to investigate the factors associated with new mucosal injuries.

## 2 Animals, Materials, and Methods

### 2.1 Study Design and Animals

This prospective study was approved by the ethics committee of our institution (approval number: 210406‐7). Consent was obtained from the owners before study inclusion.

Dogs that presented at JASMINE Veterinary Cardiovascular Medical Center to undergo MVR were eligible for inclusion. Dogs were excluded if they had clinical or historical evidence of esophageal disease, a history of brachycephalic obstructive airway syndrome, or if they were judged by cardiology clinicians to be too small in body size for TEE. TEE was performed during the anesthesia period to support MVR. A small probe (9T probe, tip size: 10.9 × 8.4 mm, GE HealthCare Japan, Tokyo, Japan) was used in dogs weighing < 5 kg, whereas a large probe (6VT‐D probe, tip size: 12.6 × 14.3 mm, GE HealthCare Japan) was used in dogs weighing ≥ 5 kg. In all dogs, the TEE probe was carefully inserted while monitoring the TEE images until a certain image was reached, without the use of fluoroscopy. Background information of the dogs was obtained from medical records, including breed, sex, age, body weight, body condition score, and ACVIM clinical stage [20].

### 2.2 Surgical Procedure and Transesophageal Echocardiography Probe Manipulation

The manipulation was performed by well‐trained cardiology clinicians (AT, YN, and TS) to support MVR after the induction of general anesthesia. Atropine sulfate (0.03 mg/kg, SC) was administered, and midazolam (0.3 mg/kg, IV) and fentanyl (5 μg/kg, IV) were administered as premedication. Subsequently, ketamine (5 mg/kg, IV) was administered to induce anesthesia, and endotracheal intubation was performed. Additionally, anesthesia was maintained using sevoflurane (1.0%–3.0%) intraoperatively, and perioperative analgesia was established by continuous rate infusion of fentanyl (18 μg/kg/min). Fentanyl was continuously administered at a rate of 3–5 μg/kg/min for 24 h postoperatively for pain management. The MVR procedure consisted of artificial chordae and annuloplasty using expanded polytetrafluoroethylene threads [21], performed under cardiopulmonary bypass with an activated coagulation time of > 300 s achieved by heparinization (heparin sodium, 200 IU/kg IV). Cardiac evaluation via TEE was performed three times during the procedure: before connecting the heart to the cardiopulmonary bypass as a preoperative heart evaluation, during cardiac arrest as support for the procedure (removing air at the time of cardiac closure and checking the heart condition immediately after resuscitation), and after weaning from the cardiopulmonary bypass (before extubation) as a postoperative heart evaluation. The clinicians reinserted the TEE probe for each of the three evaluation periods. Three‐dimensional imaging was performed with the 6VT‐D probe during the initial and final TEE evaluations as part of the pre‐ and postoperative assessments.

A digital stopwatch was used to monitor the duration of TEE. The stopwatch was started each time image acquisition was ongoing and was stopped when the probe was not being used, at which point the image was frozen to prevent overheating. Each probe had an autotemp shutdown feature set at 41.8°C, ensuring that if the temperature exceeded this threshold, the probe would automatically shut down to prevent overheating. When this temperature was reached, the probe was no longer capable of imaging until the temperature decreased below the predetermined temperature set by the manufacturer. The probe temperature was continuously monitored and stored within the ultrasound machine throughout the surgical procedure, with the highest temperature constantly updated and displayed in real time. At the end of the procedure, the highest temperature recorded during the entire procedure was reviewed. The ease of obtaining TEE images was categorized into three levels at the initial insertion: ‘poor’ (i.e., images had low temporal or spatial resolution necessitating invasive anteflexion or retroflexion of the probe to optimize visualization), ‘suboptimal’ (i.e., the image quality was reduced but did not interfere with procedural guidance nor required invasive probe manipulation), and ‘good’ (i.e., satisfactory images were obtained with minimal probe manipulation).

### 2.3 Evaluation of Mucosal Injury

A single observer (KK) performed endoscopy in narrowband imaging (NBI) mode (VQ‐5112C, tip diameter: 5.4 mm, Olympus Marketing Inc., Tokyo, Japan), which could clearly distinguish mucosal lesions [22, 23, 24]. Evaluation was performed before and after the surgical procedure to identify mucosal injuries: once after the induction of anesthesia and once before extubation to evaluate the occurrence of new esophageal lesions. The lesion sites were defined as oral to pharyngeal, upper esophagus, heart base, and lower esophagus. The severity of mucosal lesions was classified as ‘complex’ (intramural hematoma, mucosal laceration), ‘minor’ (petechiae, ecchymosis) [25], and ‘minute’ (the injury only visible on NBI mode). Petechiae were defined as small (pinpoint) red‐purple, non‐raised (macular), circular lesions, while ecchymosis was defined as larger confluent petechial lesions. An intramural hematoma was defined as a collection of blood in the submucosa causing a circumscribed elevated lesion. Laceration or abrasion was defined as a defect in the mucosal surface, as previously described in a human study [25].

### 2.4 Hemodynamic Changes

Hemodynamic changes were evaluated before and after the initial TEE insertion (prior to connecting the heart to the cardiopulmonary bypass). Arterial blood pressure was measured invasively by placing a catheter in the dorsalis pedis artery with a pressure transducer (DX‐300; Nihon Kohden, Tokyo, Japan) and a vital monitor (Life Scope VS; Nihon Kohden, Tokyo, Japan). Systolic, diastolic, and mean arterial blood pressures, as well as heart rate were obtained from the monitor. For data analysis, the arithmetic mean of 10 measurements was used. Additionally, a 15% change in systolic blood pressure and heart rate before and after TEE device insertion was considered clinically meaningful. No changes in anesthesia or medication were made during the measurements.

### 2.5 Echocardiography and Thoracic Radiography

The radiographs and transthoracic echocardiogram used in this study were the most recent data collected from medical records within 3 months prior to MVR. Both evaluations were performed on the same day. Vertebral heart size was measured using thoracic radiography. In the echocardiographic examination, the left atrial to aortic ratio was obtained from the right parasternal short axis of the heart base. The left ventricular end‐diastolic internal diameter normalized to body weight was obtained from a right parasternal view at the level of the chordae tendineae using the M‐mode. These measurements were performed by several well‐trained cardiology attending clinicians at the institution using an echocardiographic machine (Vivid E95; GE Healthcare Japan, Tokyo, Japan) with a 1.0–5.0‐MHz sector probe (6S‐D; GE HealthCare Japan, Tokyo, Japan).

### 2.6 Statistical Analysis

The Shapiro–Wilk test was used to assess the normality of the distribution of continuous variables, and normally distributed variables were expressed as the mean ± standard deviation (SD), while non‐normally distributed variables were expressed as the median [interquartile range, IQR]. Measurement values and baseline variables were compared between groups using Student's *t*‐test or Mann–Whitney U test. The paired *t*‐test or Wilcoxon Signed‐Rank test was used to compare the changes in blood pressure and heart rate pre‐ and post‐TEE, depending on the results of their respective normality analysis. Proportional comparisons were performed using Fisher's exact test. Univariate logistic regression analysis was performed to investigate the effect of the variable of interest on the appearance of a new mucosal injury, with the results expressed as odds ratios (ORs) and 95% confidence intervals (CIs). All statistical analyses were performed using the R software (version 4.2.2; Foundation for Statistical Computing, Vienna, Austria). Differences were considered statistically significant at *p* < 0.050.

## 3 Results

Sixty client‐owned dogs were enrolled between August 2021 and April 2022 and divided into two groups based on body weight: 45 dogs, < 5 kg; 15 dogs, ≥ 5 kg. The breeds included were Chihuahua (*n* = 31), mixed (*n* = 9), Cavalier King Charles spaniel (*n* = 5), Toy Poodle (*n* = 4), Maltese (*n* = 3), Pomeranian (*n* = 3), Miniature Schnauzer (*n* = 2), Papillion (*n* = 1), Shetland Sheepdog (*n* = 1), and Shiba (*n* = 1). The median body weight was 3.8 kg [IQR: 3.1–5.1 kg], and the mean ± SD age was 129 ± 22 months. Moreover, 39/60 dogs were male. The baseline characteristics and intraoperative findings by group are shown in Table 1. The maximum probe temperature during the procedure was higher in the ≥ 5 kg group (39.5°C ± 0.9°C vs. 40.4°C ± 1.1°C, *p* = 0.002). The ease of obtaining TEE images was ‘good’ in 57/60 dogs and ‘suboptimal’ in 3/60 dogs. No dogs had images classified as ‘poor’.

**TABLE 1:** Comparison of baseline characteristics between dogs weighing < 5 kg and ≥ 5 kg.

	< 5 kg (*n* = 45)	≥ 5 kg (*n* = 15)	*p*
Baseline characteristics			
Sex (female/male)	13/32	8/7	0.160
Age (months)	128 ± 22	133 ± 21	0.455
Weight (kg)	3.3 [2.8–4.0]	6.5 [5.9–8.0]	**< 0.001**.
BCS (underweight [1–3]/normal [4, 5]/overweight [6–9]; *n*)	5/38/2	0/12/3	0.084
ACVIM stage (*n*)			0.751
B2	16/45	7/15	
C	19/45	5/15	
D	9/45	3/15	
VHS	11.6 ± 1.1	11.9 ± 1.0	0.321
LA/Ao	2.11 [1.89–2.33]	1.95 [1.62–2.17]	0.133
LVIDDN	2.11 ± 0.33	2.09 ± 0.26	0.794
Intraoperative findings			
Total TEE probe manipulation time (sec)	1141 [921–1432]	1249 [1086–1533]	0.318
Maximum probe temperature (°C)	39.5 ± 0.9	40.4 ± 1.1	0**.002**
TEE image quality (poor/suboptimal/good)	0/3/42	0/0/15	0.732

*Note:* Bolded values indicate statistical significance.

Abbreviations: BCS, body condition score; LA/Ao, left atrial‐to‐aortic root ratio; LVIDDN, normalized left ventricular internal dimension in diastole; TEE, transesophageal echocardiography; VHS, vertebral heart size.

**FIGURE 1:**
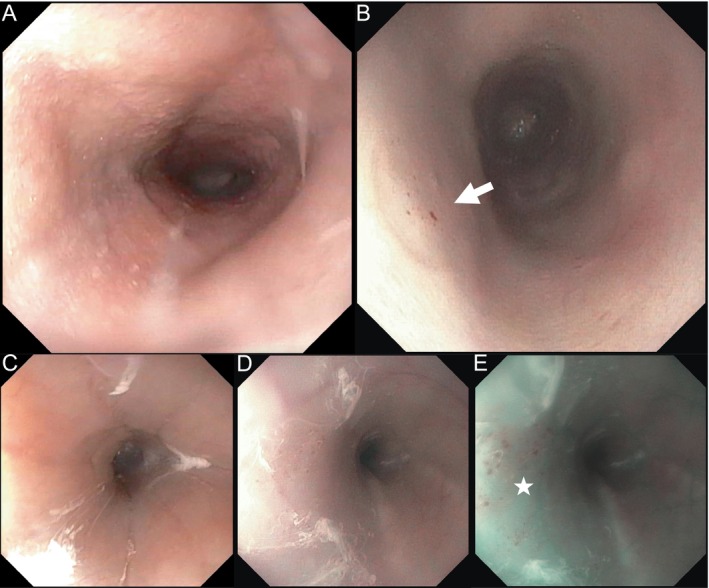
Typical esophageal mucosal findings before and after transesophageal echocardiogram. The panels show the endoscopic images of the esophagus before and after mitral valve repair. Panels A and B are images of the esophagus at the level of the heart base before and after the procedure, respectively, with a probe size of 9 T (GE Healthcare). Pinpoint mucosal petechiae are identified (white arrow). Panels C, D, and E are images of the upper esophagus before (C) and after the procedure (D, E) in a dog with a 9T probe. Panel E shows the use of a narrow‐band imaging technique at the same site. Submucosal lesions are barely visible by conventional sight (D) but are clearly observed by narrow‐band imaging (E: White star). None of the dogs showed any ‘complex’ lesions, such as intramural hematomas or mucosal lacerations.

Preprocedure, pre‐existing mucosal lesions were detected in two dogs: one presented with a single ‘minor’ lesion in the upper esophagus, and the other presented with multiple ‘minor’ lesions from the upper to the lower esophagus. These pre‐existing lesions did not worsen during the surgical procedure. Postprocedure, new mucosal lesions were detected in 20/60 dogs: 16/20 dogs had only ‘minute’ lesions, and 4/20 dogs had ‘minor’ lesions. No ‘complex’ lesions were observed (Table 2). A representative image is shown in Figure 1. Univariate logistic regression analysis identified no significant factors of new mucosal lesions for any of the variables of interest (Table 3).

**TABLE 2:** Mucosal lesions newly detected postoperatively via endoscopy.

	Total	< 5 kg	≥ 5 kg	*p*
Number of dogs with new mucosal lesion	20/60	14/45	6/15	0.75
Lesion site and severity				
Oral to pharyngeal (minute/minor/complex)	0 (0/0/0)	0 (0/0/0)	0 (0/0/0)	
Upper esophagus (minute/minor/complex)	17 (16/1/0)	12 (11/1/0)	5 (5/0/0)	
Heart base (minute/minor/complex)	2 (1/1/0)	2 (1/1/0)	0 (0/0/0)	
Lower esophagus (minute/minor/complex)	3 (1/2/0)	1 (1/0/0)	2 (0/2/0)	

*Note:* Two dogs presented with multiple lesions: one dog weighing less than 5 kg had a minute lesion at both the upper esophagus and heart base, while another dog weighing 5 kg or more had a minute lesion at the upper esophagus and a minor lesion at the lower esophagus. *p* value, Fisher's exact test between dogs weighing < 5 kg and ≥ 5 kg.

**TABLE 3:** Univariate logistic regression analysis of predictors of new mucosal lesions.

Variable	Odds ratio	95% confidence interval	*p*
TEE probe			
9 T (dogs weighing < 5 kg)	Reference		
6VT (dogs weighing ≥ 5 kg)	1.476	0.424–4.934	0.528
Age (months)	0.996	0.970–1.021	0.731
Body weight (kg)	1.008	0.775–1.277	0.948
Sex			
Female	Reference		
Male	0.722	0.237–2.240	0.566
VHS (per 0.1)	1.011	0.962–1.062	0.665
LA:Ao (per 0.1)	1.002	0.882–1.132	0.978
LVIDDN (per 0.1)	0.995	0.834–1.184	0.951
Total TEE probe manipulation time (per 100 s)	0.992	0.976–1.005	0.198
Maximum probe temperature (°C)	0.859	0.503–1.446	0.568

Abbreviations: LA:Ao, left atrial‐to‐aortic root ratio; LVIDDN, normalized left ventricular internal dimension in diastole; TEE, transesophageal echocardiography; VHS, vertebral heart size.

Regarding hemodynamic changes between before and after the first TEE insertion (preoperative heart evaluation), systolic blood pressure changed from 95 ± 13 to 92 ± 11 mmHg (*p* = 0.008), diastolic pressure changed from 56 ± 9 to 54 ± 8 mmHg (*p* = 0.063), the mean blood pressure changed from 66 ± 11 to 63 ± 9 mmHg (*p* = 0.006), and the heart rate changed from 128 ± 25 to 123 ± 24 bpm (*p* < 0.001). Overall, 44/60 of the dogs experienced a decrease in systolic blood pressure from baseline, decreasing from 99 ± 12 to 92 ± 11 mmHg (*p* < 0.001), while the remaining dogs experienced an increase from 86 ± 12 mmHg to 94 ± 12 mmHg (*p* = 0.004). The heart rate decreased from baseline in 41/60 of the dogs, falling from 131 ± 23 bpm to 121 ± 22 bpm (*p* < 0.001), while it increased in 9/60 of the dogs from 121 ± 27 bpm to 126 ± 27 bpm (*p* < 0.001). Among the dogs who showed more than a 15% hemodynamic change before and after the first TEE, 2/60 dogs had a decrease in systolic blood pressure, 5/60 dogs had a decrease in heart rate, 4/60 dogs had an increase in systolic blood pressure, and 1/60 dog had an increase in heart rate. Between the < 5 kg and ≥ 5 kg groups, there were no significant differences in pre‐TEE insertion systolic blood pressure (95 ± 13 mmHg vs. 95 ± 12 mmHg; *p* = 0.969), post‐TEE insertion systolic blood pressure (93 ± 11 mmHg vs. 90 ± 10 mmHg; *p* = 0.454), pre‐TEE insertion heart rate (127 ± 25 bpm vs. 129 ± 26 bpm; *p* = 0.833), and post‐TEE insertion heart rate (123 ± 24 bpm vs. 123 ± 25 bpm; *p* = 0.921).

## 4 Discussion

The overall incidence reported in this study is 20/60, but the severity of the lesions is considered ‘minute’ in majority of new lesions identified, even under heparin management for cardiopulmonary bypass. Although hemodynamic changes possibly occur due to TEE manipulation [4, 26], clinically meaningful changes were rarely observed in the present study. These results offer valuable insights into the safe use of TEE during cardiac surgery in veterinary clinical settings.

In human medicine, TEE‐related complications occur more frequently in pediatric humans (0.03%–6.7%) [27, 28, 29, 30, 31, 32, 33] than in adult patients (0.18%–2.8%) [34, 35, 36, 37, 38, 39]. This difference could be attributed to the small body size of pediatric patients. Dogs are similar to pediatric humans in that they generally have small body sizes. A previous study reported new esophageal lesions in 10% of dogs with a median weight of 8.7 kg [18]. The 60 dogs in the present study were smaller (median weight: 3.8 kg) than those in the above study, but only four dogs developed ‘minor’ lesions. However, the complication rate in this study was similar to that reported in the above study, despite the lower body weight. This result might be attributed to the consistent procedural approach employed for MVR. As the study focused exclusively on MVR support, the required images were mainly obtained using the mid‐esophageal four‐chamber view [4] across all dogs. We assume that different TEE views, which require further advancement and anteflexion of the probe, could lead to a higher complication rate. It is important to note that different objectives or conditions could potentially yield different outcomes.

A new technique of NBI mode endoscopy revealed lesions that were not visible with conventional views: 16/60 dogs had ‘minute’ lesions that were only visible using the NBI mode. Narrow‐band illuminations improve the contrast of the capillary pattern in the superficial layer compared to ordinary broadband illumination [40]. In humans, this technique has the potential to clearly distinguish between mucosal lesions [22, 23, 24], and improved overall accuracy for depth of superficial esophageal lesions [41]. Our results suggest that NBI can be a valuable tool for detecting subtle mucosal injuries that are not easily visible. The clinical relevance of these lesions should be further investigated in future studies.

In humans, esophageal injuries associated with TEE include ‘minor’ esophageal mucosal injuries (e.g., regions of petechiation, erosion, and hematoma), esophageal laceration and perforation, direct pressure necrosis, and thermal injury from prolonged probe contact. A study in veterinary medicine suggests that 10% and 3% of the dogs developed new lesions at the lower esophageal sphincter and at the heart base, respectively [18]. This study also reported that focal pinpoint mucosal erosion and changes in pinpoint hemorrhage were identified.

In the present study, mucosal lesions identified by endoscopy before TEE did not worsen after the procedure, and all ‘minor’ lesions were pinpoint petechiae. Furthermore, the present study found that ‘minor’ lesions were observed in one dog at the upper esophagus, one at the heart base, and two at the lower esophagus. When including minute lesions, 17/60 dogs in this study had new lesions in the upper esophagus, suggesting that the insertion and positioning of the TEE probe could have caused injury. The probe might injure the mucosa at the esophageal sphincter and upper esophagus, particularly at the curve near the thoracic inlet, where resistance can be encountered, especially in small‐breed dogs [42]. The difference in injury sites between the present study and the previous report by Stoner et al. [18], where the lesions were primarily found in the lower esophagus, can be attributed to several reasons. In the present study, TEE was inserted with retroflexion to pass the aortic arch and over an enlarged heart, while monitoring only the TEE image without fluoroscopic guidance, which could have contributed to mucosal injury during insertion. Additionally, we did not routinely obtain advanced views, such as the caudal esophageal position for a heart base view, which requires further advancement and anteflexion of the probe [4]. Most importantly, the ‘minute’ lesions observed in the upper esophagus using NBI imaging had not been previously visualized. Moreover, the ‘minor’ lesions found in the lower esophagus in this study were consistent with those reported in the previous study [18].

The authors hypothesized that small breed dogs with cardiac enlargement could compress the thoracic space and have a shorter distance from the heart to the vertebra [19], potentially increasing the risk of TEE‐related mucosal injury. However, in the present study, body weight, VHS, LA:Ao, and LVIDDN were not identified as significant risk factors for the development of new mucosal lesions. Based on these results, TEE was considered safe even in small‐breed dogs with cardiac enlargement, at least in the context of performing MVR surgery.

As previously described in human medicine, a prolonged duration of TEE might be a risk factor for mucosal injury [43]. However, our findings did not reveal a relationship between TEE active imaging time and mucosal injury. As this study focused solely on surgical support for MVR, the manipulation times among dogs were relatively consistent. A more comprehensive evaluation and continuous procedural support with TEE, particularly for congenital heart diseases such as patent ductus arteriosus and pulmonary vulve stenosis [18], would likely require additional time and extensive probe manipulation, which could lead to complications. Additionally, there was no difference in manipulation time between the dogs weighing < 5 kg with a 9T probe and the dogs weighing ≥ 5 kg with a 6VT‐D probe. However, the maximum temperature of the 6VT‐D probe was higher than that of the 9T probe, possibly because of the three‐dimensional imaging performed with the 6VT‐D probe. Nevertheless, the probe temperature might not have influenced the findings in this study, as few lesions were identified at the heart base, and the probe temperature did not increase the probability of identifying new mucosal lesions, similar to findings in human medicine [25].

In humans, possible gastrointestinal complications following TEE include odynophagia, dysphagia, hoarseness, and nausea [28, 36, 37, 44]. In the present study, various factors influenced postoperative gastrointestinal symptoms [45, 46] making it difficult to determine whether TEE findings are specifically associated with reflux or vomiting after surgery. Additionally, nausea without vomiting is challenging to assess in dogs. Under general anesthesia, the use of sevoflurane, as was used in this study, can be associated with postoperative regurgitation and vomiting [46, 47]. Particularly during cardiopulmonary bypass, hemodilution and anemia can occur due to overhydration from the bypass circuit fluid and cardioplegia solution [48], which could contribute to postoperative gastrointestinal complications and transfusion‐related gastrointestinal signs [46, 49]. Further research is required to clarify the gastrointestinal symptoms associated with TEE‐related mucosal injury. With respect to hemodynamic changes, blood pressure and heart rate can decrease during TEE.

A report conducted 24‐h Holter monitoring and blood pressure measurements in 54 unsedated human patients undergoing TEE, suggesting that the average systolic blood pressure increased from 125 to 141 mmHg in 77% of the patients, whereas 22% experienced a decrease from 122 to 115 mmHg during the examination [50]. However, it is important to note that these findings are based on outcomes in unsedated humans.

In contrast, a decrease in systolic blood pressure was more common in the present study of anesthetized dogs, with 41/60 dogs showing a decrease and only 9/60 dogs showing an increase. Given that this study was conducted under anesthesia, which mitigated the stress associated with TEE probe manipulation, a procedure‐related increase in blood pressure was avoided. The mild decrease in blood pressure might have been due to the vagus nerve reflex triggered by esophageal stimulation [51, 52]. In cats, TEE probe compression can induce occlusion of pulmonary venous inflow, potentially resulting in cardiac arrest and bradycardia [26].

We believe that the clinically meaningful hemodynamic changes were minimized in this study. However, it should be noted that these changes likely depend on factors such as baseline blood pressure, probe selection, and body size. While no dogs received changes in medication or anesthesia during the initial TEE examination, controlling baseline hemodynamic values proved challenging and might have influenced the results. Indeed, dogs with increased systolic blood pressure tended to have lower baseline systolic blood pressure, and vice versa. A similar trend was observed with heart rate. A few dogs exhibited more than 15% hemodynamic changes before and after TEE, likely due to vagal stimulation or heart compression, highlighting the need for close monitoring of potential bradycardia and hypotension throughout the procedure.

### 4.1 Study Limitations

This study has some limitations. First, TEE was primarily used for intraoperative management, necessitating minimal imaging. Therefore, the dogs in our study did not require invasive manipulation of the probe to obtain images, which might limit the generalizability of our findings into other clinical settings. Second, the total time the probe remained in the esophagus was not measured in this study; however, as the probe was removed after each measurement, this does not appear to be a substantial issue. The assessment of mucosal lesions was inherently subjective, introducing potential variability into the findings. Additionally, the clinical impact of minute mucosal lesions remains unclear and warrants further investigation in other clinical settings. In terms of hemodynamic changes, it was challenging to strictly control baseline values in the clinical setting due to factors such as anesthesia depth and ventilator settings. Finally, the conclusions of this study are based on a relatively small sample size and limited dog breeds, which could restrict the generalizability of the results. Further investigation in different types of surgeries and dog breeds is necessary.

## 5 Conclusions

During MVR in dogs, TEE‐related mucosal injuries were relatively rare. The results suggest that TEE can be safely performed in small‐breed dogs with cardiac enlargement, even with heparin administration. Although slight changes in blood pressure and heart rate were observed, these changes might not be solely attributable to TEE. Careful monitoring of hemodynamic parameters during TEE probe manipulation is recommended.

## Disclosure

Authors declare no off‐label use of antimicrobials.

## Ethics Statement

Approved by the Institutional Ethics Committee of JASMINE Veterinary Cardiovascular Medical Center (approval number: 210406‐7). Authors declare human ethics approval was not needed.

## Conflicts of Interest

The authors declare no conflicts of interest.
